# Successful treatment of a mixed candidal and bacterial skull base osteomyelitis with antimicrobial agents and hyperbaric oxygen therapy—A rare case report

**DOI:** 10.1002/kjm2.12939

**Published:** 2025-01-15

**Authors:** Yu‐Hsin Liu, Chun‐Chieh Wu, Yen‐Hsu Chen, Chun‐Yu Lin

**Affiliations:** ^1^ Division of Infectious Diseases, Department of Internal Medicine Kaohsiung Medical University Hospital, Kaohsiung Medical University Kaohsiung Taiwan; ^2^ Department of Pathology Kaohsiung Medical University Hospital, Kaohsiung Medical University Kaohsiung Taiwan; ^3^ School of Medicine, Graduate Institute of Medicine, College of Medicine Center for Tropical Medicine and Infectious Disease Research, Kaohsiung Medical University Kaohsiung Taiwan; ^4^ School of Medicine, College of Medicine National Sun Yat‐Sen University Kaohsiung Taiwan

Skull base osteomyelitis (SBO) has very high morbidity and mortality, which usually affects elderly patients with diabetes.^1^ The most prevalent pathogenic microorganism is *Pseudomonas aeruginosa*, and therapy of this bacterium is becoming more difficult as reports of antimicrobial resistance.^2^ Fungi are very rare pathogens of SBO. In a retrospective cohort study, hyperbaric oxygen therapy (HBOT) effectively treated advanced SBO.^3^ Herein, we report a rare case co‐infected with candidal and bacterial SBO managed successfully with combined antimicrobial agents and HBOT.

A 57‐year‐old male with poorly controlled diabetes mellitus (DM) and psoriasis vulgaris without immunosuppressants presented with a complaint of a severe headache and left otalgia for 3 months. A brain computed tomography (CT) scan at a local hospital revealed a mass over the left Rosenmuller fossa. Magnetic resonance imaging revealed no central nervous system involvement. Pathology of the nasopharynx biopsy showed no malignancy. After being discharged from the local hospital without a diagnosis or treatment, fever, general malaise, and drowsy consciousness continued. One week later, drowsiness, dysphagia, and slurred speech were found. After admission to our hospital, pleocytosis with monocytes predominant (Cell count: 173/μL, PMN/monocytes: 14%/86%) was seen in cerebrospinal fluid. Acyclovir 500 mg every 8 h was initially administered for suspected aseptic meningitis. Ceftazidime combined with amikacin was administered for suspected malignant external otitis.

A brain CT scan at our hospital further found bony erosion over the clivus bone, and other findings were consistent with infection/inflammatory process, and osteomyelitis with perilesional spreading in the left aspect of the skull base (Figure 1C). Surgical biopsy and debridement were used for the mass lesion from the nasopharynx. Bacterial culture revealed *P. aeruginosa*. Besides, pathology examination revealed inflammatory cell infiltration into bony tissue, as well as invasive candidiasis, including some entrapped candida yeast and pseudohyphae by periodic acid‐Schiff (PAS) staining (Figure 1A,B). Based on mixed invasive fungal infection with osteomyelitis of the skull base and meningitis, amphotericin B was also added for 2 weeks, and subsequently changed to fluconazole due to hypokalemia and hypomagnesemia.

**FIGURE 1:**
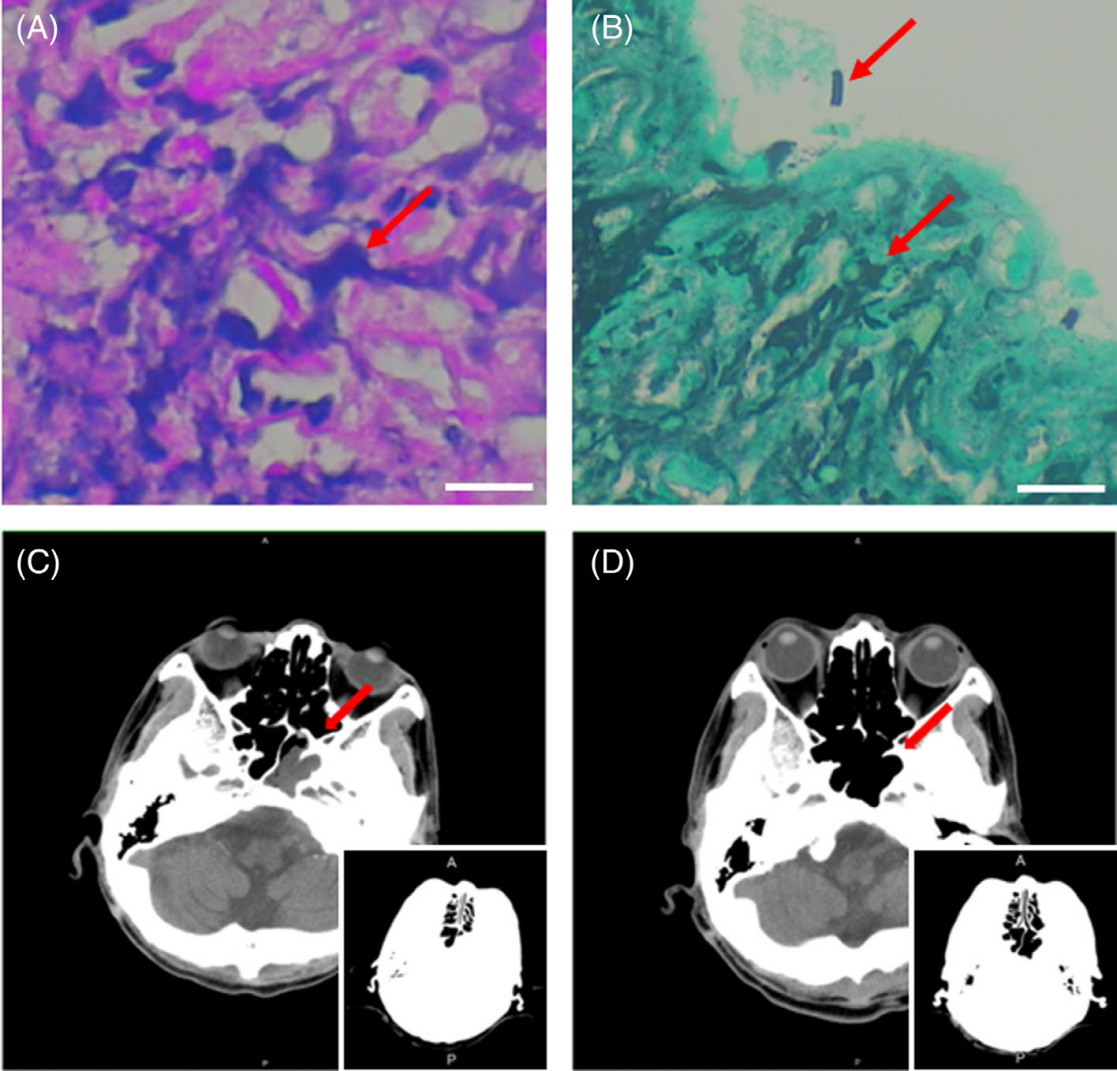
(A) Pseudohyphae on PAS staining as dark blue (red arrow) against a pink background (scale bar = 10 μm). (B) Pseudohyphae on Giemsa staining as black (red arrow) against a green background (scale bar = 10 μm). (C) Brain CT before treatment showed osteomyelitis with perilesional spreading in the left aspect of the skull base, involving the left aspect of the nasopharynx and left lower portion of the left cerebellopontine angle. (D) After the operation, antimicrobial agents, and hyperbaric oxygen therapy, follow‐up brain CT showed much improvement with an 8‐month interval. The right lower panels in (C) and (D) displayed the bone window pictures of the corresponding images.

To decrease infected tissue edema and improve blood flow to the affected area, HBOT was initiated 4 weeks after diagnosis of mixed fungal and bacterial SBO. Ceftazidime was changed to ciprofloxacin, according to the culture report. After the patient became stable, he was discharged with oral ciprofloxacin and fluconazole, and continued outpatient HBOT. The erythrocyte sedimentation rate (ESR) decreased significantly after the first 30 HBOT sessions. His clinical state and imaging results improved after another 30 HBOT sessions. CT scan 6 months after discharge revealed retropharyngeal pre‐vertebral soft tissue thickening and mild demineralization of the clivus (Figure 1D). Neurological symptoms also improved after treatment. As IDSA guideline recommendation for the treatment duration of candida osteomyelitis by fluconazole is 6–12 months, also due to sub‐optimally controlled DM, slowly improved inflammatory markers, such as ESR and C‐reactive protein (CRP) levels, as well as the residual lesions and continuous improvement of CT scan upon following up, ciprofloxacin was given for 6 months and fluconazole for 1 year. No neurological sequelae affected his daily life, and 10 years of follow‐up showed no incidence.

This rare case of polymicrobial, life‐threaten SBO reminded the effectiveness of HBOT in addition to combined antimicrobial agents, in agreement with several previous studies.^3^ Sometimes tissue cultures cannot yield fungi. However, recent studies suggest that 16S amplicon sequencing may improve the earlier pathogen identification and the prescription of precision therapy.^4^, ^5^

## CONFLICT OF INTEREST STATEMENT

The authors declare no conflict of interest.

## Data Availability

The data that support the findings of this study are available from the corresponding author upon reasonable request.
